# *Aeromonas* spp. and Traveler’s Diarrhea: Clinical Features and Antimicrobial Resistance

**DOI:** 10.3201/eid0905.020451

**Published:** 2003-05

**Authors:** Jordi Vila, Joaquin Ruiz, Francisco Gallardo, Martha Vargas, Lara Soler, Maria José Figueras, Joaquin Gascon

**Affiliations:** * Hospital Clinic, Barcelona, Spain; † Universitat Rovira i Virgili, Reus, Spain; ‡ Universitat de Barcelona, Barcelona, Spain

**Keywords:** *Aeromonas*, diarrhea, travellers, research

## Abstract

Traveler’s diarrhea is the most common health problem of international travelers. We determined the prevalence of *Aeromonas* spp. associated with traveler’s diarrhea and analyzed the geographic distribution, clinical features, and antimicrobial susceptibility. *Aeromonas* spp. were isolated as a cause of traveler’s diarrhea in 18 (2%) of 863 patients. *A. veronii* biotype sobria was isolated in nine patients, *A. caviae* in seven patients, and *A. jandai* and *A. hydrophila* in one patient each. *Aeromonas* spp. were isolated with a similar prevalence in Africa, Latin America, and Asia. Watery and persistent diarrhea, fever, and abdominal cramps were common complaints. All strains were resistant to ampicillin; showed variable resistance to chloramphenicol, tetracycline, and cotrimoxazole; and were susceptible to cefotaxime, ciprofloxacin, and nalidixic acid. The persistence of symptoms made antimicrobial treatment necessary.

Traveler’s diarrhea is the main infectious disease reported in persons traveling abroad. Among the microorganisms responsible, bacteria represent approximately 61% (*1*). Enterotoxigenic *Escherichia coli*, enteroaggregative *E. coli,* and *Shigella* spp. are the most common bacteria involved (*1*,*2*). Other bacteria that cause diarrhea, such as *Salmonella, Campylobacter, Yersinia, Aeromonas,* and *Plesiomonas* spp., are isolated less often (*1*).

The genus *Aeromonas* has high diversity: at least 16 DNA hybridization groups are recognized (*3*). Among these genospecies, *A. hydrophila, A. caviae,* and *A. veronii* biotype sobria are considered of clinical significance (*4*,*5*). The spectrum of infectious diseases caused by *Aeromonas* species includes gastrointestinal infections as well as extraintestinal infections such as cellulitis, wound infections, septicemia, urinary tract infections, and hepatobiliary and ear infections, among others (*6*). Although healthy carriers of *Aeromonas* spp. have been described, several case-control studies have shown that these bacteria cause diarrhea (*6*). With the incorporation of genotypic techniques, identification of *Aeromonas* to species level has improved (*7*). The main objective of this study was to determine the prevalence of *Aeromonas* spp. as a cause of traveler’s diarrhea and to analyze the species’ geographic distribution, clinical features, and susceptibility to antimicrobial agents.

## Methods

### Patients

A total of 863 patients with traveler’s diarrhea were recruited from the Tropical Medicine Unit of the Hospital Clinic of Barcelona, Spain, during the period January 1999–December 2001. All patients completed an epidemiologic questionnaire; their clinical history was taken, and a physical examination was performed. Traveler’s diarrhea was defined as the occurrence of three or more episodes of watery stool within a 24-hour period, with or without other symptoms, or the occurrence of unformed stools accompanied by one of the following: vomiting, nausea, abdominal cramps, fever, chills, prostration, or tenesmus. Persistent diarrhea was defined as that of >14 days’ duration.

### Microbiologic Tests

A stool sample was collected, sent to the Laboratory of Clinical Microbiology, and processed for bacterial, viral, and parasitologic studies. To isolate *Aeromonas* spp., blood agar supplemented with ampicillin and a selective media, CIN (cefsulodin-irgasan-novobioci) agar, were used. After incubation at 37°C for 24–48 hours, an oxidase test was performed on the colonies compatible with bacilli. Biochemical criteria were used to identify *Aeromonas.* Identification of the species was performed by 16S rDNA–restriction fragment length polymorphism as previously described (*7*,*8*). The biotype of *A. veronii* strains was identified on the basis of a positive reaction to arginine dihydrolase and negative response to bilis-esculin hydrolysis and production of ornithine decarboxylase (*9*).

### Susceptibility Testing

Antimicrobial susceptibility tests were performed by using an agar disk diffusion method advocated by the National Committee for Clinical Laboratory Standards (*10*). Antimicrobial disks (ampicillin 10 μg; cefotaxime 30 μg; chloramphenicol 30 μg; ciprofloxacin 5 μg; nalidixic acid 30 μg; tetracycline 30 μg; and trimethoprim-sulfamethoxazole 1.25/23.75 μg) were obtained from Becton Dickinson (Cockeysville, MD). *E. coli* ATCC 25922 and *Pseudomonas aeruginosa* ATCC 27853 were used as quality-control strains.

## Results

### Distribution and Geographic Origin of Species Causing Traveler’s Diarrhea

*Aeromonas* spp. were isolated as a cause of traveler’s diarrhea in 18 (2%) of 863 patients. *A. veronii* biotype sobria was isolated in nine patients, *A. caviae* in seven patients, and *A. jandaei* and *A. hydrophila* in one patient each (Table 1). In three of these patients, another enteropathogen was also found: in one patient who had traveled to Mexico, *Shigella sonnei* was isolated together with *A. veronii*; in another patient traveling to India, *Giardia lamblia* was detected together with *A. veronii*; in the third patient, who had traveled to Thailand, *Salmonella* Typhimurium was found with *A. veronii*. The frequency of *Aeromonas* spp. as a cause of traveler’s diarrhea was similar in patients returning from Africa (1.7%), Latin America (1.8%), and Asia (2.3%) (Table 1).

**Table 1:** Species and geographic distribution of clinical isolates of *Aeromonas* spp. causing traveler’s diarrhea

Geographic area	*A. veronii* biotype sobria n=9	*caviae* n*=7*	jandai n=1	*A. hydrophila* n=1
Guatemala	1	1		
India	2	1^a^	1^a^	1
Iran		1		
Kenya	1			
Mali/Burkina Faso	1			
Mexico	2^b^			
Nicaragua		1		
Paraguay		1		
Sahara	1			
Senegal		2		
Thailand	1			

^a^This patient traveled to India and Nepal. ^b^One traveler also visited Guatemala.

### Clinical Features

The signs and symptoms of *Aeromonas* enteritis in these 18 patients are summarized in Table 2. Sixteen of the 18 patients had watery diarrhea; these were the cases associated with *A. veronii* biotype sobria and *A. caviae.* The patients with enteritis caused by *A. hydrophila* and *A. jandaei* had loose stools. Fifty percent of the patients had fever and abdominal cramps, whereas nausea and vomiting were uncommon complaints. Gross blood was observed in the stools of one patient, but this could be attributed to the *S. sonnei* isolated in the same stool. In 9 of the 18 patients, diarrhea was persistent.

**Table 2:** Clinical features of patients with traveler’s diarrhea associated with *Aeromonas* spp.

	No. of patients with symptoms/total patients (n=18)		
Sign or symptom	*A. veronii* biotype sobria	*A. caviae*	Overall^a^
Watery diarrhea	9/9	7/7	16/18
Abdominal cramps	6/9	3/7	10/18
Persistent diarrhea	3/9	5/7	9/18
Fever	6/9	3/7	10/18
Nausea, vomiting, or both	2/9	0/7	3/18
Gross blood in stools	1^b^/9	0/7	1/18

^a^Also includes *A. hydrophila* and *A. jandai.* ^b^In this patient, a *Shigella sonnei* strain was also isolated.

### Antimicrobial Susceptibility

The antimicrobial susceptibility of *Aeromonas* spp. isolates causing traveler’s diarrhea is shown in Table 3. All strains were resistant to ampicillin but susceptible to cefotaxime, ciprofloxacin, and nalidixic acid. The susceptibility to chloramphenicol, tetracycline, and trimethoprim-sulfamethoxazole varied. Some 66.6% of *A. veronii* biotype sobria strains and 71.4% of *A. caviae* strains were susceptible to chloramphenicol; 55.6% of *A. veronii* biotype sobria strains and 71.4% of the *A. caviae* strains were susceptible to tetracycline; and 77.8% of *A. veronii* biotype sobria and 100% of *A. caviae* were susceptible to trimethoprim-sulfamethoxazole.

**Table 3:** Antimicrobial susceptibility of *Aeromonas* spp. causing traveler’s diarrhea

	No. of isolates showing susceptibility	
Antimicrobial agent	*A. veronii* biotype sobria (n=9)	*caviae* (n=7)
Ampicillin	0	0
Cefotaxime	9	7
Chloramphenicol	6	5
Ciprofloxacin	9	7
Nalidixic acid	9	7
Tetracycline	5	5
Trimethoprim/sulfamethoxazole	7	7

### Treatment

While travelling, two patients received treatment, amoxicillin in one case and amoxicillin plus clavulanic acid in the other. Patients with persistent diarrhea were treated with the following antibiotics: norfloxacin (one patient), ciprofloxacin (six patients), and trimethoprim-sulfamethoxazole (two patients); all recovered.

## Discussion

In this study we describe the prevalence of different types of *Aeromonas* species associated with traveler’s diarrhea in a cohort of travelers to a variety of tropical and subtropical countries. In contrast, other published studies have often been selective in terms of the types of travelers, geographic areas visited, or attempts to isolate specific microorganisms with the aim of testing antibiotic efficiency. In our study, *Aeromonas* spp. were isolated in 18 (2%) of 863 patients with traveler’s diarrhea. *A. veronii* biotype sobria and *A. caviae* were the most frequently isolated species. These findings agree with the results of Hänninen et al. (*11*), who reported that these were the most common *Aeromonas* spp. associated with traveler’s diarrhea in tourists traveling to Morocco. Likewise, Yamada et al. (*12*) found that *A. veronii* biotype sobria was the *Aeromonas* species most frequently implicated as a cause of traveler’s diarrhea in Japanese travelers returning from unindustrialized countries. In our study, the geographic distribution of *Aeromonas* species did not favor any predominant area: species were isolated with a similar prevalence in Africa, Latin America, and Asia. However, all four species (*A. veronii* biotype sobria, *A. caviae, A. jandaei,* and *A. hydrophila)* were isolated from patients returning from India. In India, *Aeromonas* spp. has been identified as an enteric pathogen in 1.8% of patients with diarrhea (*13*). In a recent study performed in Dhaka (Bangladesh), *Aeromonas* spp. were significantly associated with diarrhea, similar to occurrences in other countries (*14*–*17*).

In our study, 3 (16.7%) of the *Aeromonas* isolates were detected together with other enteropathogens. This situation allowed us to consider that the symptoms we observed in the patients with traveler’s diarrhea associated with *Aeromonas* spp. were due to the presence of this *Aeromonas* organisms. In our study, watery stools, fever, and abdominal cramps were the most common symptoms, which is consistent with other reports (*11*,*18*). Albert et al. (*18*) suggested that isolates of *Aeromonas* spp. positive for both the *alt* and *ast* genes, which encode enterotoxins, were associated with watery diarrhea but that isolates positive only for the *alt* gene were associated with loose stools.

Fifty percent of the patients with *Aeromonas* spp. enteritis had persistent diarrhea. Chronic diarrhea lasting more than 1 year caused by *A. caviae* has been reported (*2*). A direct link between drinking water and food contaminated with *Aeromonas* spp. and gastrointestinal disease has been demonstrated (*19*).

Patients with prolonged enteritis required treatment. A quinolone was the drug of choice, although increased occurrence of quinolone-resistant *Aeromonas* spp. strains has been reported in industrialized countries (*20*,*21*). Regarding the β-lactam antibiotics, *Aeromonas* spp. strains analyzed in this study were, as expected, uniformly resistant to ampicillin, whereas third-generation cephalosporins, such as cefotaxime, showed good activity. These results are in accordance with those reported by other authors, showing that third-generation cephalosporins are active against *Aeromonas* spp (*2*,*22*). The percentage of strains with resistance to chloramphenicol, tetracycline, or trimethoprim-sulfamethoxazole ranged from 22.9% to 45%. These levels of resistance are likely related to the extensive use of these antimicrobial agents in unindustrialized countries.

In summary, *A. veronii* biotype sobria and *A. caviae* are the *Aeromonas* species most frequently associated with traveler’s diarrhea; watery diarrhea, fever, and abdominal cramps are the predominant clinical features. The persistence of symptoms makes the use of antimicrobial treatment necessary.
